# Fiber Bragg Grating Measuring System for Simultaneous Monitoring of Temperature and Humidity in Mechanical Ventilation

**DOI:** 10.3390/s17040749

**Published:** 2017-04-02

**Authors:** Carlo Massaroni, Michele A. Caponero, Rosaria D’Amato, Daniela Lo Presti, Emiliano Schena

**Affiliations:** 1 Unit of Measurements and Biomedical Instrumentation, Center for Integrated Research, Università Campus Bio-Medico di Roma, Via Álvaro del Portillo, 21, 00128 Rome, Italy; c.massaroni@unicampus.it (C.M.); lp.daniela@yahoo.it (D.L.P.); 2 Photonics Micro- and Nanostructures Laboratory Research Centre of Frascati, ENEA, Via Enrico Fermi, 45, Frascati, 00044 Rome, Italy; michele.caponero@enea.it (M.A.C.); rosaria.damato@enea.it (R.D.)

**Keywords:** fiber optic sensors, fiber Bragg grating sensors, humidity measurement, temperature measurement, moisture-sensitive polymer, mechanical ventilation

## Abstract

During mechanical ventilation, the humidification of the dry air delivered by the mechanical ventilator is recommended. Among several solutions, heated wire humidifiers (HWHs) have gained large acceptance to be used in this field. The aim of this work is to fabricate a measuring system based on fiber Bragg grating (FBG) for the simultaneous monitoring of gas relative humidity (RH) and temperature, intended to be used for providing feedback to the HWHs’ control. This solution can be implemented using an array of two FBGs having a different center wavelength. Regarding RH monitoring, three sensors have been fabricated by coating an FBG with two different moisture-sensitive and biocompatible materials: the first two sensors were fabricated by coating the grating with a 3 mm × 3 mm layer of agar and agarose; to investigate the influence of the coating thickness to the sensor response, a third sensor was developed with a 5 mm × 5 mm layer of agar. The sensors have been assessed in a wide range of RH (up to 95%) during both an ascending and a subsequent descending phase. Only the response of the 3 mm × 3 mm-coated sensors were fast enough to follow the RH changes, showing a mean sensitivity of about 0.14 nm/% (agar-coated) and 0.12 nm/% (agarose-coated). The hysteresis error was about <10% in the two sensors. The contribution of temperature changes on these RH sensors was negligible. The temperature measurement was performed by a commercial FBG insensitive to RH changes. The small size of these FBG-based sensors, the use of biocompatible polymers, and the possibility to measure both temperature and RH by using the same fiber optic embedding an array of two FBGs make intriguing the use of this solution for application in the control of HWHs.

## 1. Introduction

During physiological ventilation, the inspired air is warmed and humidified by the upper airways, and reaches the carina at a systemic temperature and saturation (37 °C and RH of 100%) [1,2]. During invasive ventilation, the upper airways are bypassed, so the humidification of the dry air delivered by the mechanical ventilator is recommended to every patient. HWHs have gained large clinical acceptance for providing this missing heat and moisture to the gases delivered to mechanically ventilated patients [3,4]. The use of HWHs is strongly recommended in invasive ventilation [1]. Their performances are essential to have good clinical outcomes because inadequate humidification can cause several side effects, such as: atelectasis, a worsening of the lung compliance, and an increase in local susceptibility of bacterial invasion, impairment of mucociliary clearance, as well as a water condensation within the breathing circuit with a consequent increment of airflow resistance and incidence of infections [5,6]. Basically, HWHs consist of a humidification chamber containing water that is leaned on a metallic plate, which is heated by Joule effect. The chamber is placed along the inspiratory limb of the breathing circuit, between the mechanical ventilator and the patient. When the gas delivered by the ventilator passes through the chamber, it exchanges heat and moisture with the water. The heat loss on the metallic plate regulates the water temperature, and thus the thermo-hygrometric conditions of the gas at the chamber outlet. Most commercial HWHs implement a simple strategy to adjust the heat loss on the metallic plate: the heat loss on the plate is adjusted in order to keep constant the temperature at the chamber outlet (usually at 37 °C), that is measured by a thermocouple or a thermistor. A number of factors influence the phenomena of heat and moisture exchange within the chamber, therefore the mentioned control strategy does not allow for an appropriate way to manipulate the heat loss on the metallic plate. The consequence is inadequate gas conditioning. Indeed, the performance of HWHs are influenced by environmental conditions (e.g., temperature room) and by ventilatory settings (e.g., minute volume, tidal volume), as shown in literature [7,8,9,10,11].

Several sensors have been developed to monitor the RH, mainly based upon electrical (i.e., capacitive or impedance-type) or mechanical (i.e., strain-effects and mass-loading effects) transducers. Recently, fiber optic-based sensors have been gaining interest in the measure of this parameter [12]: they are particularly appreciated for their performance in harsh environments (i.e., chemical inertia, electromagnetic immunity), high sensitivity, small size, light weight and also remote analysis capability. Lastly, optical fibers are bio-compatible, so they are gaining acceptance in biomedical applications [13].

A number of RH sensors based on fiber optics have been proposed, including the use of in-fiber gratings, evanescent wave techniques, interferometric methods, hybrid approaches and absorption methods, extensively reviewed in [14,15]. In particular, the in-fiber grating sensors represent a class of intrinsic fiber optic sensors that has gained widespread popularity in recent years mainly because of their sensitivity to environmental parameters such as temperature and strain [16]. Depending on the grating structure of the sensing element, in-fiber grating sensors can be classified in FBG and Long Period Grating (LPG) [17,18].

For humidity sensing purposes, the strain sensitivity of the FBG has been employed as the underlying sensing mechanism: the hygroscopic expansion of the coating induces strain in the fiber core and relative wavelength shift in the FBG signal that can be collected and processed. Several coatings have been investigated, such as silica/di-ureasil [19], polyimide [20,21,22,23,24], polyvinyl alcohol [25,26,27], thermoplastic polyimide [28], and Pyralin [20].

The aim of this paper is to develop an FBG-based system able to measure both the gas temperature and the RH at the chamber outlet, in order to pave the way for new and more performant strategies for HWHs’ control. The temperature measurement is simply performed by a commercial FBG, that is almost insensitive to RH due to the acrylate coating. Regarding RH monitoring, we fabricated and tested three FBG-based sensors: two sensors were coated by a 3 mm × 3 mm-layer (the first one with agar and the second one with agarose), and the third was coated by a 5 mm × 5 mm-layer of agar. Some solutions have been proposed to fabricate RH fiber optic sensors using agarose: (i) optical fiber humidity sensors based on the deposition of agarose on biconically tapered optical fiber have been investigated in [29,30]. Basically, they are intensity-modulated sensors, where the light intensity is related to the refractive index change of the agarose that is induced by RH. In [29] experiments were carried out for RH ranging between 30% and 80%, and showed a reversible sensor response with a small hysteresis and a maximum optical output dynamic range of 6.5 dB. Although the quite short rise time (about 5 s) makes the sensor suitable for RH monitoring at the HWH chamber outlet, the sensor response was not investigated under near-saturation conditions that usually occur at the chamber outlet. A similar configuration was proposed in [30] in the range of 50% to 80%, showing a linear response of the sensor; (ii) agarose has also been used to surround an LPG sensor. In this solution the resonance wavelength of the LPG was modulated by the RH-induced refractive index change of the agarose [31]. The sensitivity was 114.7 pm/%, but the resonance wavelength was only sensitive to higher RH values (i.e., 70%–95%); (iii) Other optical systems are based on photonic crystal fiber interferometer (PCFI) infiltrated with agarose [32]. The refractive index of the agarose infiltrated into the micro-holes of the PCF modulates the reflected optical power.

A system able to perform a simultaneous measurement of humidity and temperature for application in mechanical ventilation has been also proposed. The system was based on a film of bilayers of Poly(allylamine hydrochloride) (PAH) and silica (SiO_2_) nanoparticles [33]. They used an FBG sensor for temperature monitoring (sensitivity of 10 pm∙°C^−1^); the RH was measured by an ad hoc fabricated film acting as a Fabry-Perot cavity (sensitivity of −1.4 × 10^−12^ W/%). Finally, several solutions based on coated-LPG for RH measurements have been reported using different materials, such as agarose, silica nano-spheres, polyimide, and hydrogel [31,34,35,36]. Although the principle of work is similar to the sensor based on a uniform FBG, the output signal of LPG is modulated by both strain and refractive index changes caused by the hygroscopic coating.

Therefore, there are not investigations focused on agarose- or agar-coated FBG sensors for RH measurements.

The use of agar or agarose (which is obtained by agar) has several advantages: preparation and coating stages are easy, and they have a wide range of operation in terms of RH values, so that several applications prefer to base the fiber optic sensors’ coating on this polymeric component [37,38]. Moreover, these two materials are organic and biocompatible coatings, therefore they can be used in the application of our interest, where they will be placed within the breathing circuit and hit by the medical gases delivered to patients. Lastly, this solution is intrinsically electric safe, avoiding risks when inflammable gases (e.g., pure oxygen) are delivered to patients.

## 2. Working Principle and Fabrication of the Sensors

### 2.1. Working Principle of the Sensors

The working principle of the proposed RH sensor is based on two main pillars: the FBG sensitivity to strain and the attitude of the hygroscopic materials to absorb water vapor with a consequent increase in volume.

Regarding the FBG sensors, they can be seen as a notch filter which reflects back a narrow light spectrum centered around a wavelength, termed the Bragg wavelength (λ_B_), that satisfies the Bragg condition [39]. The value of λ_B_ is considered the output of these gratings in the most sensing applications, and can be expressed as:

(1) $$\lambda_{B}=2\cdot \Lambda \cdot \eta_{\mathrm{eff}}$$

where Λ is the spatial period of the grating, and η_eff_ is the effective refractive index of the fiber optic. These two parameters are the function of strain and temperature, therefore FBG sensors have gained popularity in medical applications for temperature and strain sensing [13]. The Bragg wavelength shift (Δλ_B_) induced by either a strain (ε) or a temperature change (ΔT) is expressed by the following equation:

(2) $$\frac{\Delta \lambda_{B}}{\lambda_{B}}=\left(1-P_{e}\right)\cdot \varepsilon +\left[\left(1-P_{e}\right)\cdot \alpha +\xi \right]\cdot \Delta T$$

where P_e_, α, and ξ are the photoelastic constant, the thermal-expansion coefficient, and the thermo-optic coefficient of the optical fiber, respectively.

**Figure 1:**
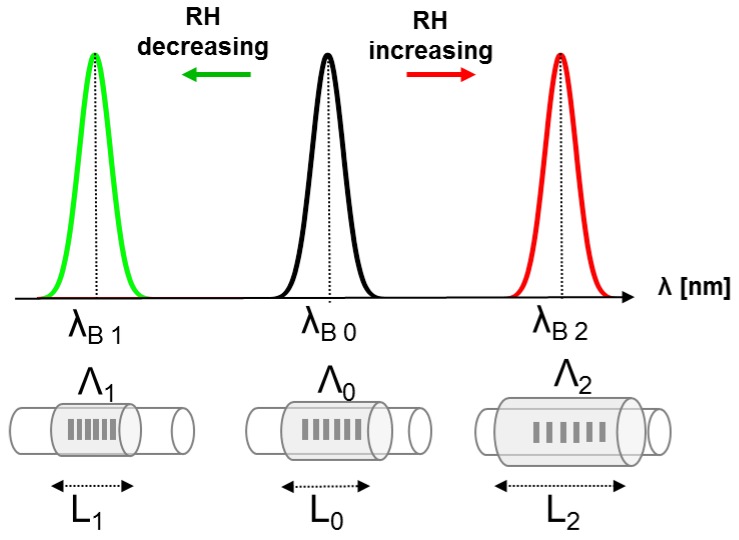
The image shows the lengths and the spatial periods of the gratings, as well as the Bragg wavelengths at three different RH values. The RH variation induces the volumetric expansion (when RH increases) or contraction (when RH decreases) of the hygroscopic material, resulting in the increase of both its length (L_2_) and of the spatial period of the grating (Λ_2_) when RH increases, or in the decrease of both its length (L_1_) and of the spatial period of the grating (Λ_1_) when RH decreases.

The sensitivity of the FBG output to RH is obtained by coating the FBG with a hygroscopic material; the principle of work can be explained by two steps, as represented in Figure 1: (i) an increment of environmental humidity causes the adsorption of water molecules that pass through the agarose or agar pores, replacing the air. This phenomenon is underpinned by the hydrophilic nature of these two materials and causes the swelling of the agarose or agar layer. The higher the environmental humidity, the bigger the swelling of these coatings; (ii) some hygrosensitive materials (agarose and agar among them) possess a good adhesion to silica, so that their swelling causes microstrain in the FBG with a consequent Δλ_B_, that can be considered an indirect measure of RH.

Regarding the temperature monitoring, it is performed by using a commercial FBG sensor coated by acrylate, which is sensitive to this parameter as shown in Equations (1) and (2).

### 2.2. Fabrication of the Sensors

Among a number of possible hygroscopic coating materials, agar and agarose have been chosen because they satisfy the following criteria that make them suitable for monitoring RH in the breathing circuit during artificial ventilation: (i) they offer a wide range of operation for RH measurement, considering the high RH values encountered in mechanical ventilation; (ii) they have a low tendency to evaporate and should exhibit good long term stability. This feature is important because the ventilation can also last weeks or months; (iii) they are biocompatible; (iv) the coating procedure is easy; and (v) they possess a good adhesion to silica.

Agar is composed of two main mixed substances: agarose (the gelling fraction) and agaropectin. Agarose usually represents two-thirds of the natural agar, and consists of repeating units of D-galactose and L-galactose.

The double helical structure of the gelling fraction aggregates form a 3-D supporting structure that holds the water molecules. In this way, a gel with several valuable features (i.e., with sharp melting and sharp gelling points, rigid, brittle, and with well-defined shapes) is formed. Concentration, sugar content, and time, as well as pH strongly influence the gel strength. Agar and agarose are soluble in hot water and the solution forms a gel on cooling to about 35–45 °C that does not melt below 85 °C.

To fabricate the RH sensor, an ad hoc designed steel mold with a hairline notch in the middle was fabricated to perform the coating of the FBG with agar and agarose (Sigma-Aldrich). The commercial FBG (Broptics Technology Inc., Taipei, Taiwan; 1 cm length of sensing area) was fixed straight and horizontally in the middle of the notch and then coated with hot (>85 °C) solution. The solutions were prepared by dissolving 10 wt % agar or 1.5 wt % agarose in distilled water at 100 °C using a heater combined with a magnetic stirrer. The mixture was deposited on the optical fiber when the solution was at temperature higher than the gelling point.

The temperature measurement was performed by a commercial FBG (Broptics Technology Inc., Taipei, Taiwan; 1 cm length of sensing area). In order to have a negligible cross-sensitivity to RH, an FBG coated by acrylate was selected (commercial FBGs coated by polyimide are more sensitive to RH because this coating is hygroscopic). The temperature changes during the experiments were calculated by considering the thermal sensitivity of the sensors, that is 0.01 nm∙°C^−1^, as reported by the manufacturer (Broptics Technology Inc., Taipei, Taiwan).

## 3. Experimental Set-up and Results

In this section the experimental set-up used to assess the response of the proposed RH sensors to a wide range of RH values and the data collected during the experiments are shown. The output changes of the FBG used for temperature monitoring are also shown.

### 3.1. Description of the Experimental Set-up

**Figure 2:**
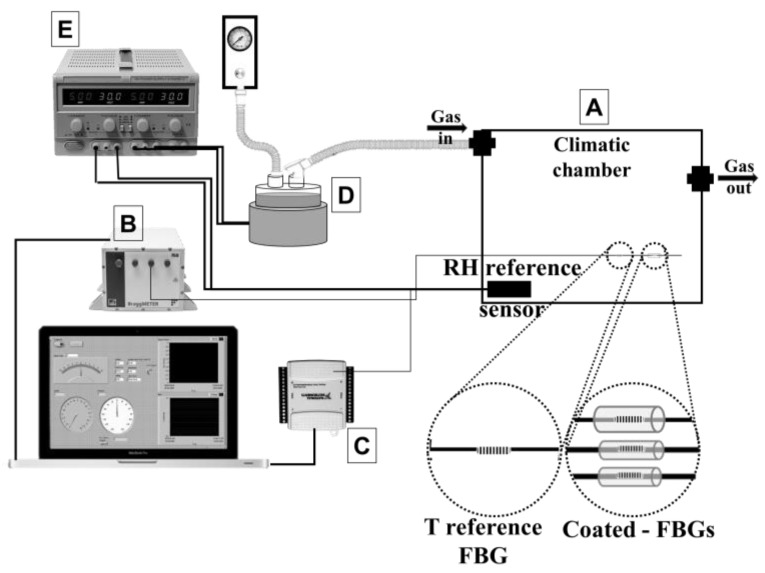
Experimental set-up: (**A**) climatic chamber; (**B**) fiber Bragg grating interrogator system; (**C**) data acquisition board (DAQ NI USB-6009); (**D**) heated wire humidifier; (**E**) DC power supply.

The three RH sensors under investigation are placed within a climatic chamber (Figure 2A) that is conditioned by an airflow delivered by pressurized sources and adjusted by a knob. The output of the three RH sensors and the output of an FBG used for temperature measurement is collected by a fiber Bragg grating interrogator system (FS22, HBM Fiber Sensing, Portugal; wavelength measurement range: from 1500 nm to 1600 nm, resolution = 1.0 pm, Figure 2B) at a frequency of 1 Hz. A capacitive humidity sensor (HIH 4602A, Honeywell International Inc., Morristown, NJ, USA; range of measurement: from 0% to 100%, accuracy = ±3.5%) is used to provide the RH reference value. The voltage output of this sensor is converted to a digital signal by a data acquisition board (DAQ NI USB-6009, National Instruments, Figure 2C) at a sample frequency of 50 Hz; then it is converted to an RH value by using the input-output relationship provided by the manufacturer. This analysis is performed in LabVIEW environment, by using an ad hoc developed Virtual Instrument.

The experimental set-up is able to test the sensor in a wide range of RH: indeed, it allows drying the air within the plastic box up to RH = 5%–10% or humidifying the air up to RH = 100%. When the aim is to apply low or decreasing RH values, the dry air coming from the pressurized sources directly flows to the climatic chamber. When the aim is to apply high or increasing RH values, the air is firstly conditioned and then is delivered to the chamber. The air conditioning is carried out by forcing the air to flow into a humidification chamber of a commercial HWH (MR850, Ginevri S.r.l., Rome, Italy, Figure 2D). The RH value at the chamber outlet is controlled by manually adjusting the heat loss on the heater plate of the HWH using a DC power supply (IPS2302A, ISO-TECH, Figure 2E).

The output of the commercial FBG insensitive to RH can be used with a twofold aim: to monitor the gas temperature during the experiments, and to compensate the output changes of the three RH sensors due to temperature changes.

### 3.2. Results

**Figure 3:**
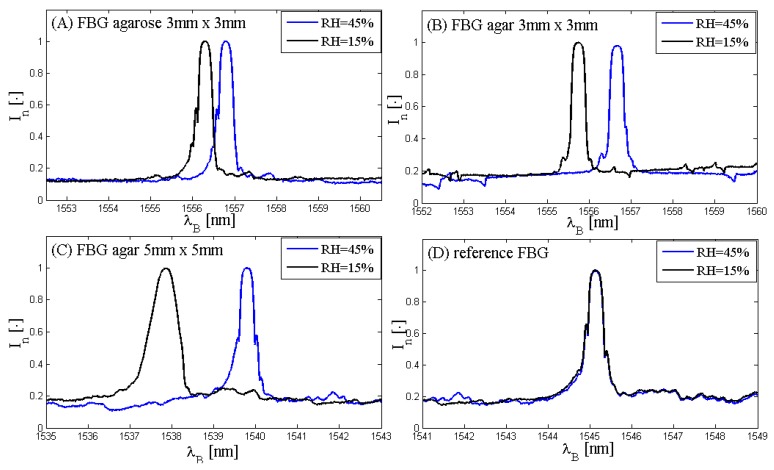
Spectra of the three fiber Bragg grating (FBG)-coated sensors and of the reference FBG sensor at different RH values: (**A**) spectra of the agarose 3 mm × 3 mm-coated FBG at RH = 15% (black line) and RH = 45% (blue line); (**B**) spectra of the agar 3 mm × 3 mm-coated FBG at RH = 15% (black line) and RH = 45% (blue line); (**C**) spectra of the agar 5 mm × 5 mm-coated FBG at RH = 15% (black line) and RH = 45% (blue line); (**D**) spectra of the reference FBG at RH = 15% (black line) and RH = 45% (blue line).

Firstly, the spectra of the three FBG-coated sensors and the spectrum of the reference FBG have been acquired in order to assess the spectra characteristics and to figure out if the spectra shift with RH changes. It is expected that the spectra of the three FBG-coated sensors shift toward the longer wavelength when RH increases, otherwise the reference sensor may be almost insensitive to RH changes. This explorative analysis has been performed at two different values of RH (about 15% and about 45%) and at an almost constant temperature (about 20 °C). Figure 3 shows these results.

Figure 3 shows that the spectra of the three coated FBGs (Figure 3A–C) are similar to the reference sensor (Figure 3D). Furthermore, the spectra of the three FBG-coated sensors shift toward the bigger wavelength with RH increase due to the expansion of the coating material; on the contrary, the spectrum shift of the reference FBG is almost negligible, which is consistent with the hypothesis of negligible sensitivity to RH.

In order to investigate if the proposed sensors are able to follow RH changes in a wide range of values, a cycle consisting of two phases has been applied. Specifically, the phases are: (i) ascending RH levels, starting from dry air (RH < 10%) to almost saturation condition (RH > 95%). It lasted about 20 min; (ii) descending RH levels, starting from the end of the first phase (RH > 95%) to almost dry air (RH < 15%). It lasted about 12 min.

**Figure 4:**
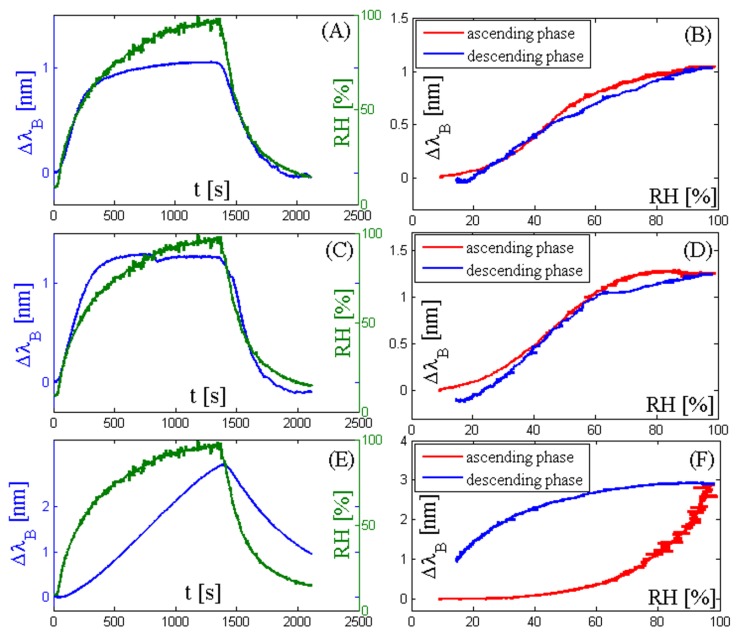
(**A**) RH trend during the cycle (green line) and corresponding Bragg wavelength shift of the sensor output (blue line) coated by agarose (3 mm × 3 mm); (**B**) Bragg wavelength shift of the sensor output coated by agarose (3 mm × 3 mm) vs. the RH values during both the ascending phase (red line) and descending phase (blue line); (**C**) RH trend during the cycle (green line) and corresponding Bragg wavelength shift of the sensor output (blue line) coated by agar (3 mm × 3 mm); (**D**) Bragg wavelength shift of the sensor output coated by agar (3 mm × 3 mm) vs. the RH values during both the ascending phase (red line) and descending phase (blue line); (**E**) RH trend during the cycle (green line) and corresponding Bragg wavelength shift of the sensor output (blue line) coated by agar (5 mm × 5 mm); (**F**) Bragg wavelength shift of the sensor output coated by agar (5 mm × 5 mm) vs. the RH values during both the ascending phase (red line) and descending phase (blue line).

Figure 4A,C show that the response of the two 3 mm × 3 mm-coated sensors is able to follow the changes of RH as fast as the reference sensor or faster, whose response time in slow moving air is 50 s according to the data provided by the manufacturer. On the other hand, the response of the 5 mm × 5 mm-coated sensor (Figure 4E) is too slow and the sensor is not able to follow the RH changes. The trend of the input-output relationship of the two 3 mm × 3 mm sensors is quite similar (Figure 4B,D). The maximum Δλ_B_ is about 1.0 nm for the agarose-coated sensor and 1.2 nm for the agar-coated sensor. These two sensors followed the RH changes, so their mean sensitivity can be estimated as the ratio between the Δλ_B_ recorded in correspondence to the two extreme RH values applied during the test and the difference between these two RH values. The sensitivity is about 0.14 nm/% and 0.12 nm/% for agar and agarose 3 mm × 3 mm-coated sensors, respectively. The degree of hysteresis for the two 3 mm × 3 mm-coated sensors is acceptable (i.e., 10% and 8% for agar-coated and agarose-coated, respectively). However, it is too high for the 5 mm × 5 mm-coated sensor (about 81%) due to its very slow response.

**Figure 5:**
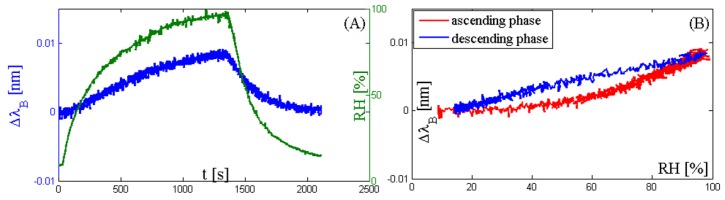
(**A**) RH trend during the cycle (green line) and corresponding Bragg wavelength shift of the temperature sensor (blue line); (**B**) Bragg wavelength shift of the temperature sensor output as a function of the RH values during both the ascending phase (red line) and descending phase (blue line).

The temperature increment (ΔT) estimated by the reference FBG during the cycle is reported in Figure 5.

Figure 5A shows that the maximum Δλ_B_ is experienced at the end of the ascending phase; this is the result of a slight temperature increment of the gas within the climatic chamber during this phase. Indeed, the gas passes through the humidification chamber of the HWH, where it absorbs vapor and also experiences a slight temperature increment. Considering the thermal sensitivity of the sensor (i.e., 0.01 nm∙°C^−1^), the maximum temperature increment experienced within the climate chamber is about 1 °C. Figure 5B provides information about the Δλ_B_ during the two phases, but it does not represent the sensitivity of this sensor to RH, because the Δλ_B_ is caused by the temperature increment. Finally, we want to point out that this temperature increment can be considered negligible in the analysis of the response of the three RH sensors because the Δλ_B_ caused by RH changes (Figure 4) is much bigger (two orders of magnitude) than the one caused by the temperature change (Figure 5).

## 4. Discussion and Conclusions

In this paper, we fabricated and tested three polymer-coated FBG sensors for RH measurements. Moreover, a further FBG insensitive to RH (with a coating layer made of acrylate) was used for temperature gas monitoring. RH measurements show that both agar- and agarose-coated FBGs have a good sensitivity to RH in a wide range of values. Specifically, the 3 mm × 3 mm-coated sensors have a sensitivity bigger than 0.1 nm/%; moreover, they are able to follow both ascending and descending phases of RH covering a wide range of values within about 20 min and 12 min, respectively. The 5 mm × 5 mm-coated sensor is more sensitive but its response is too slow to follow the mentioned phases of RH. According to the purpose of this work, too slow response times are unacceptable for breathing monitoring because damage caused by under-humidification has a dynamic faster than 20 min [40]. This makes preferable for our aims the 3 mm × 3 mm-coated sensors. Indeed, in mechanical ventilation it is pivotal to heat and moisten the air delivered by the ventilator. HWHs have gained popularity in this application [2]. The simple control strategy, based on maintaining constant the gas temperature at the outlet of the humidification chamber, is not always able to provide an optimal gas conditioning [9].

The aim of this work is to develop an FBG-based system for the simultaneous monitoring of gas RH and temperature at the humidification chamber outlet; these measures are intended to be used as feedback to improve the control strategy of the HWH. The sensors must satisfy strict criteria considering that: they will be used at high RH values, because at the chamber outlet the gas is often under near-saturation conditions; the system must be electric safe, because the patients are often ventilated with inflammable gas (i.e., pure oxygen); it must be small-sized, because it will be placed within the breathing circuit (~2 cm of inner diameter) and should not add a significant airflow resistance to the respiratory system; it must be biocompatible, because the gas that hits the sensors is delivered to critically ill patients; the RH sensor must show low degradation, because some patients are continuously ventilated for weeks or months. The use of FBGs coated by agar or agarose for RH monitoring and by acrylate for temperature monitoring succeeds in meeting all these features. The humidity changes-based ∆λ_B_ are due to the swelling and deswelling of the hygroscopic material (i.e., agar or agarose) in contrast to the typical behavior of acrylate-coated fiber optic sensors, which are in general insensitive to humidity. Moreover, agar and agarose are hygroscopic and show good adhesion to silica, and their coating procedure is easy. We want to point out that the use of agar is motivated by its low cost with respect to agarose. Lastly, this solution allows monitoring both temperature and RH by inserting within the breathing circuit only one fiber optic, that embeds two FBGs.

Among several optical fiber humidity sensors, FBG-based sensors have been largely investigated. Yeo et al. [24] proposed a system whose sensitivity was enhanced by using a sensing polymer layer of polyimide that induced a substantial strain effect on the FBG (sensitivity of ~3.0 × 10^−3^ nm/%). An interesting solution was proposed by Chen et al. [41], who fabricated a system for monitoring both the RH and the temperature by using a polyimide-coated FBG for the RH measurement and an acrylate-coated one for the temperature. The humidity sensor sensitivity was around 2.6 × 10^−3^ nm/%. The use of poly(methyl methacrylate) (PMMA) used by Zhang et al. to coat FBGs showed a linear response of the sensor to the RH variations with a sensitivity of about 35.2 pm/% [42]. The sensitivity dependence on the coating thickness was also investigated by Kronenberg et al. in [21], who found an increase in sensitivity corresponding with the polyimide coating thickness. These data agree with the ones obtained in the present study: the 5 mm × 5 mm agar-coated FBG shows the maximum wavelength shift due to the biggest volumetric expansion of the coating compared to the 3 mm × 3 mm agar- and agarose-coated sensors, at the expense of the response time and of the degree of hysteresis (~81%), see Figure 4. The 3 mm × 3 mm agarose- and agar-coated FBGs show a mean sensitivity of about 0.12 nm/% and 0.14 nm/% in the whole testing range, respectively. These values are slightly bigger than the sensitivity found in [24,41]. Hysteresis analysis demonstrated acceptable values for the two 3 mm × 3 mm-coated sensors (<10% in both cases), slightly higher than the ones reported by Yeo et al. (i.e., 5% in the range 23%–97%) [23] and Li et al. (i.e., ~4%) [26]. Although several groups of research proposed fiber optic-based systems for simultaneous measurements of temperature and humidity using different principles of work, only Hernandez and coauthors [33] focused their attention on the measurements of these parameters in mechanical ventilation. The monitoring of these parameters is pivotal to improve the HWHs’ humidification process, since their performance is influenced by air temperature, humidity and other ventilatory settings, as reported in literature [7,8,43,44,45]. Among previous works, Hromadka et al. [46], Urrutia at al. [47], Liu et al. [48], and Viegas et al. [34] calibrated their humidity sensors based on LPG up to RH values of 70%, 80%, 75%, and 80%, respectively; Wu et al. performed the calibration of their Fabry-Perot interferometry-based humidity sensor up to 90% [49]. Therefore, their sensor cannot be used in mechanical ventilation (often the RH at the chamber outlet is >95%). Only the work proposed by Sun et al. [50] and by Arregui et al.[51] reported a calibration up to higher values of RH (95% and 97%, respectively), but their sensor showed a pronounced nonlinearity at these high values.

The proposed solution uses biocompatible materials, it is electric safe, and it permits performing both temperature and RH monitoring by using only a single and small fiber optic within the breathing circuit without adding a substantial resistance increase to the respiratory system of the patients. Although further experiments will be performed to investigate the sensor response during long-lasting experiments with gas under near-saturation conditions, the promising mentioned features make this investigation the premise to develop a new system, for improving the control strategy of HWHs and their performances.
